# Joint coordinate attention mechanism and instance normalization for COVID online comments text classification

**DOI:** 10.7717/peerj-cs.2240

**Published:** 2024-08-19

**Authors:** Rong Zhu, Hua-Hui Gao, Yong Wang

**Affiliations:** 1 School of Computer Science, Qufu Normal University, Rizhao, China; 2 Laboratory Experimental Teaching and Equipment Management Center, Qufu Normal University, Rizhao, China

**Keywords:** Coordinate attention mechanism, Instance normalization, Text classification, Gaussian Error Linear Unit, COVID online reviews

## Abstract

**Background:**

The majority of extant methodologies for text classification prioritize the extraction of feature representations from texts with high degrees of distinction, a process that may result in computational inefficiencies. To address this limitation, the current study proposes a novel approach by directly leveraging label information to construct text representations. This integration aims to optimize the use of label data alongside textual content.

**Methods:**

The methodology initiated with separate pre-processing of texts and labels, followed by encoding through a projection layer. This research then utilized a conventional self-attention model enhanced by instance normalization (IN) and Gaussian Error Linear Unit (GELU) functions to assess emotional valences in review texts. An advanced self-attention mechanism was further developed to enable the efficient integration of text and label information. In the final stage, an adaptive label encoder was employed to extract relevant label information from the combined text-label data efficiently.

**Results:**

Empirical evaluations demonstrate that the proposed model achieves a significant improvement in classification performance, outperforming existing methodologies. This enhancement is quantitatively evidenced by its superior micro-F1 score, indicating the efficacy of integrating label information into text classification processes. This suggests that the model not only addresses computational inefficiencies but also enhances the accuracy of text classification.

## Introduction

Text classification has been explored in various fields such as sentiment analysis (Zainuddin, Selamat & Ibrahim, 2018) and questions and answers (Gweon & Schonlau, 2024). In the age of information explosion, manually processing and categorizing large amounts of text data is both time-consuming and challenging. In addition, the accuracy of manual text categorization is easily affected by human factors. Text classification has been used for several years; however, the classification methods have focused on input text manipulation. Traditional text classification models dominate, such as Plain Bayes (NB) (Xu, 2018), K-nearest neighbors (KNN) (Alhutaish & Omar, 2015) and support vector machines (SVM) (Wan et al., 2012). Later, deep neural networks such as convolutional neural networks (CNNs) (Malik & Jain, 2024; Zhu, 2021) and recursive neural networks (Li et al., 2020) proved more effective in text encoding. Subsequently, Bidirectional Encoder Representations from Transformers (BERT) (Sung, Park & Kim, 2023), XLNet (Wu, Wang & Zhao, 2023), and other large pre-training models achieved substantial performance improvements owing to their powerful coding capabilities. However, these methods usually rely on highly differentiated text representations and require significant computational resources.

To mitigate resource limitations, this study leverages label information to address such challenges. In single-cell research (Qu, Kao & Hakonarson, 2024), scientists aim to uncover the details of cellular heterogeneity and dynamic changes through high-resolution analysis of individual cells. This field has rapidly progressed due to advancements in high-throughput sequencing technologies, enabling researchers to measure and analyze gene expression in single cells. This leads to a deeper understanding of cell types, states, and functions. A key objective in single-cell research is to identify and classify different cell types, which is crucial for comprehending the composition and function of complex biological systems, such as human tissues or tumors. To achieve this, researchers have developed various algorithms and tools to extract meaningful features from single-cell RNA sequencing data for cell classification and annotation.

Single-cell research is crucial because it allows scientists to study the gene expression of individual cells, revealing cellular heterogeneity and dynamic changes that are not detectable in bulk cell analyses. This detailed understanding is vital for identifying and classifying different cell types, understanding their functions, and discovering new cell states. This is important for various aspects of biological research, including developmental biology, disease mechanisms, and regenerative medicine. In connection to RNA studies, single-cell RNA sequencing (scRNA-seq) enables precise measurement of RNA molecules in individual cells, providing insights into gene expression patterns and regulatory mechanisms at an unprecedented resolution. This helps in understanding the complexity of biological systems, identifying biomarkers, and developing targeted therapies.

Wu et al. (2023) focused on the label information between cells to better extract features, which also inspired text classification tasks. In a text classification task, the role of labels is to capture more relevant words during classification. Wang et al. (2018) developed an attention model known as LEAM, which integrated the label and word vectors into the same space through construction using label embeddings. Du et al. (2019) added an interactive mechanism to the process of text classification to enable the model to obtain the corresponding word-matching signals in the classification process.

Experiments confirmed that the above model maintained good performance under the premise of a simpler architecture and fewer parameters. However, the attention adopted by the model considers only the effects of text labels. Embedded label information was not fully used. Therefore, this study further combines the attention from text to label, integrates text and label, and makes the model look for more labels to match the text in the process of encoding label embedding. In comparison with previous label embedding methods, the fusion method of text and labels makes full use of the feedback information of text representations and encodes this feedback into labels.

Deep-learning models involving attention mechanisms (Zhang & Wu, 2023) have been used in classification and chromosome science. The coordinate attention mechanism (Lu et al., 2016; Yu et al., 2017; Lu et al., 2019) is widely used in multi-channel learning between images and languages. Lu et al. (2016) applied a coordinate attention model to image inference. Recently, to enhance the learning of image content, Lu et al. (2019) adopted a coordinate attention transformer to embed images and text. The study referenced in Vaswani et al. (2017) proposes a novel architecture entirely based on attention mechanisms, designed to replace traditional recurrent neural networks and convolutional neural networks. This methodology facilitates the generation of mutually attentive representations, enabling the explicit capture of relationships between text and labels. Liu et al. (2022) adopted the IN method within a self-attention model, subsequently applying an activation function to enhance the model’s functionality. This modification not only provides stochastic regularization but also markedly augments the overall performance of the model. Our model is built on the basis of this framework.

This research introduces a classification algorithm predicated on a coordinate attention mechanism enhanced by IN (Ulyanov, Vedaldi & Lempitsky, 2016). The model comprises two principal components: an IN Text and Label Coordinate Attention Encoder (INTLCE) and an Adaptive Label Decoder (ALD). The INTLCE is engineered to generate interactive representations that elucidate the interconnections between texts and labels. Conversely, the ALD is designed to delineate and apprehend the correlations amongst labels. The salient contributions of this study are delineated as follows: (1) The proposed model integrates text and label information, thereby augmenting the utilization of label data through the synergistic fusion of text and labels, which underscores the model’s innovative approach to considering the symbiotic relationship between text and label information. (2) Enhancing the conventional coordinate attention mechanism, this study incorporates IN and GELU functions. These additions are aimed at augmenting model efficacy while concurrently mitigating training challenges and computational exigencies. (3) The model’s efficacy was assessed employing the COVID online comments dataset, wherein it demonstrated competitive performance relative to contemporaneous studies utilizing the identical dataset. This evaluation underscores the model’s robustness and its potential applicability to real-world datasets.

## Coordinate attention mechanism model with IN method

**Figure 1:**
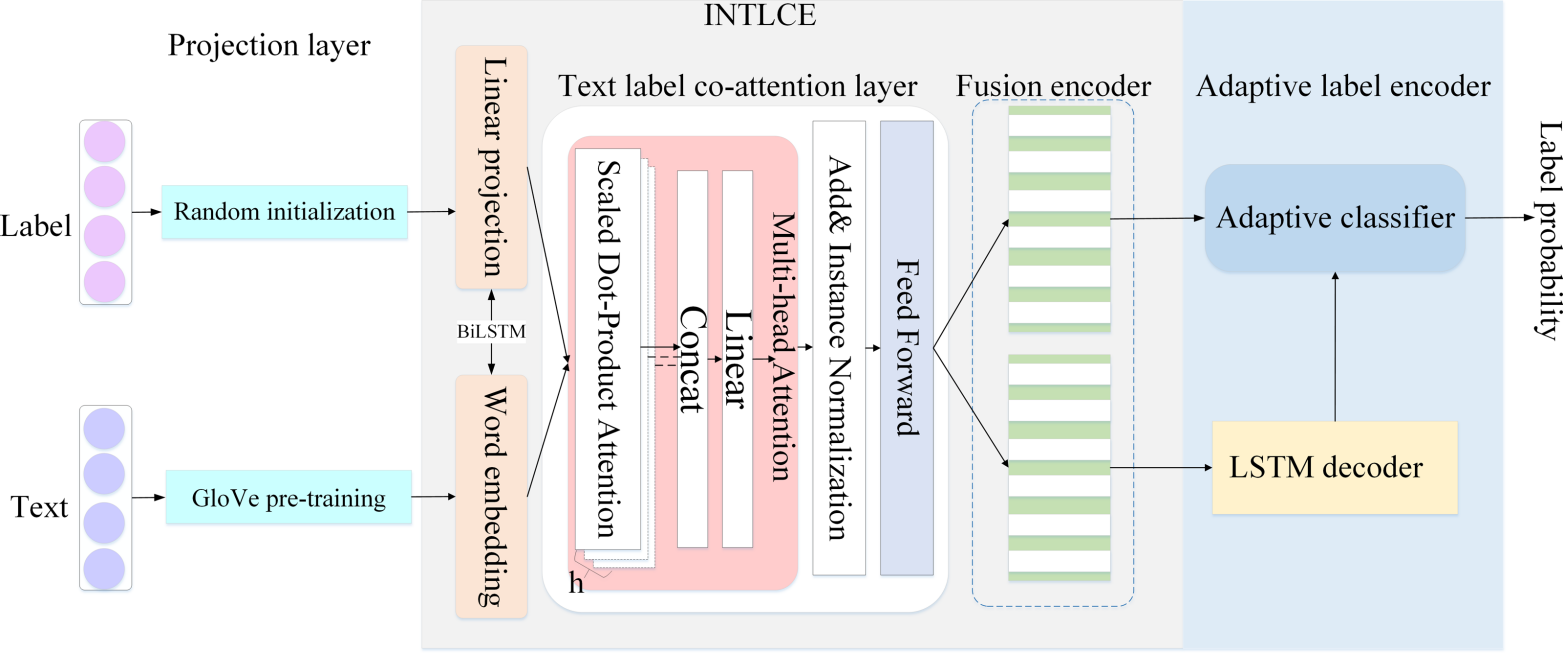
Model architecture.

We proposed an optimized coordinate attention network. First, we unified the encoding and embedding of labels and text. Thereafter, the IN method was used to accelerate the convergence ability of the model and reduce the complexity of training. Finally, their common participation representations were used to generate the target labels. The structure of the model is shown in Fig. 1.

In this study, the label generation problem was applied to text classification. Specifically, given the text *x* , our goal was to generate probability $\hat {y}$ for all categories.

### Overall plan

Let a text sequence with *m* words be represented as $x= \left[ {x}_{1},\ldots ,{x}_{m} \right] $, and let the set of labels be $\wp = \left\{ {l}_{1},\ldots ,{l}_{c} \right\} $, where *c* denotes the total number of categories (such as the binary classification used in this study, *c* = 2). The labels are divided into negative and positive categories, which prepare the model for learning and final validation. Note that the text sequence *x* in the document contains *m* words, and the label sequence *l* in document contains ℘ labels. The mapping process is shown in the following equation. The label are associated with the note text denoting *z*_*text*_, and the label with the note text denoting *z*_*label*_.

\begin{eqnarray*}{z}_{text},{z}_{label}={f}_{enc} \left( x,l \right) , \end{eqnarray*}

where *f*_*enc*_ represents the process of encoding a mapping function. After entering the two representations of *z*_*text*_ and *z*_*label*_ obtained using above formula, we use the decoder to generate the probability sequence $\hat {y}$ as follows:

\begin{eqnarray*}\hat {y}={f}_{dec} \left( {z}_{text},{z}_{label} \right) , \end{eqnarray*}

where *f*_*dec*_ represents the process of decoding the mapping function. Using the above equation, the decoder can use the mutual representation of the text and labels to make a final prediction.

### INTLCE

In this section, we discuss the INTLCE module in detail. INTLCE encodes text and label sequences into mutually participating labels and text representations. Specifically, INTLCE is divided into the projection, text label coordinates attention and fusion encoder layers.

We used the bidirectional long short term memory (BiLSTM) (Graves & Schmidhuber, 2005) approach to effectively encode words and labels and enhance our understanding of input sequences. In addition, this approach helps identify correlations between labels, which further improves the accuracy of the encoding process.

Given a sequence of words and labels *x* ∈ *R*^*m*^ and *l* ∈ *R*^*c*^, we start by mapping words and labels separately to the word-embedding layer *x*_*emb*_ ∈ *R*^*m*×*d*_*emb*_^ and label-embedding layer *L*_*emb*_ ∈ *R*^*c*×*d*_*emb*_^ respectively, where *d*_*emb*_ denotes the embedding dimension.

For each sentence, we used pre-trained GloVe word embedding (Kamyab, Liu & Adjeisah, 2021; Ibrahim et al., 2021). This is an unsupervised word-vector representation technique. The resulting representation depicts the linear substructure in the word vector space by training the aggregated global word-word co-occurrence statistics in the corpus.

For each label, a random initialization was used for embedding. To improve computational efficiency, we first used an independent linear projection layer. Suppose *x*_*enb*_ and *L*_*emb*_ are projected separately into a more compact, smaller-dimensional embedding, where *X*_*proj*_ ∈ *R*^*m*×*d*^, *L*_*proj*_ ∈ *R*^*c*×*d*^, *d* < *d*_*emb*_, *d* is a hidden dimension.

Thereafter, we used the BiLSTM method for word embedding *X*_*proj*_ ∈ *R*^*m*×*d*^ and label embedding *L*_*proj*_ ∈ *R*^*c*×*d*^ in the projection layer. The calculation equations are expressed as follows:

\begin{eqnarray*}\begin{array}{@{}l@{}} \displaystyle {X}_{enc}=BLM \left( {X}_{proj} \right) ,\\ \displaystyle {L}_{enc}=BLM \left( {L}_{proj} \right) , \end{array} \end{eqnarray*}

where BLM is short for BiLSTM, *X*_*enc*_ denotes text encoding and *L*_*enc*_ is the label coding. In our implementation, we incorporated weight sharing into the BiLSTM. This means that the same weights are used for both the forward and backward directions of the network, which can help reduce the number of parameters and improve efficiency.

Furthermore, the study introduces a novel normalization technique, termed IN, which represents an innovative approach to the standardization of data within the experimental framework. This method enhances the analytical rigor by ensuring consistency in data treatment, thereby improving the reliability and interpretability of the results. IN is independent of the channel or batch size and ensures the independence of each text instance, which enhances the performance of the model. Another optimization approach involves replacing the original self-attention mechanism activation function with the GELU functions. Random regularization was added to make the model more consistent with the cognitive processes.

First, the key steps in optimizing the coordinate attention mechanism focus on the scaled dot product attention, which is expressed as follows:

\begin{eqnarray*}Attention \left( Q,K,V \right) =Softmax \left( \frac{Q{K}^{T}}{\sqrt{{d}_{h}}} \right) V, \end{eqnarray*}

where *Q* ∈ *R*^*q*×*d*_*k*_^, *K* ∈ *R*^*k*×*d*_*k*_^, and *V* ∈ *R*^*v*×*d*_*v*_^. The multi-head attention equation is expressed as follows:

\begin{eqnarray*}MHead \left( Q,K,V \right) =Softmax \left( \left[ {H}_{1};...;{H}_{p} \right] \right) {W}^{0}, \end{eqnarray*}

where ${H}_{i}=Attention \left( Q{W}_{i}^{Q},K{W}_{i}^{K},V{W}_{i}^{V} \right) $, and the projection parameters are ${W}_{i}^{Q}\in {R}^{{d}_{k}\times {d}_{p}}$, ${W}_{i}^{K}\in {R}^{{d}_{k}\times {d}_{p}}$, ${W}_{i}^{V}\in {R}^{{d}_{v}\times {d}_{p}}$, and ${W}_{i}^{O}\in {R}^{p{d}_{p}\times {d}_{h}}$. We used *d*_*k*_ = *d*_*v*_ = *d*_*p*_ and *d*_*p*_ = *d*_*h*_/*p* as the dimensions of each head, where *d* represents the dimension of the interval model, *p* represents the number of heads, and $ \left[ \cdot \right] $ represents the join operation.

Next, because the traditional self-attention mechanism (Vaswani et al., 2017) only considers text modes, the matrices *Q*, *K*, and *V* represent text encodings. The text code *X*_*enc*_ and label code *L*_*enc*_ were simultaneously input into multiple attention modules, and the self-attention module was converted into a coordinate attention module.

\begin{eqnarray*}\begin{array}{@{}l@{}} \displaystyle {X}_{att}=MHea{d}_{X} \left( {X}_{enc},{L}_{enc},{L}_{enc} \right) ,\\ \displaystyle {L}_{att}=MHea{d}_{L} \left( {L}_{enc},{X}_{enc},{X}_{enc} \right) , \end{array} \end{eqnarray*}

where *X*_*att*_ ∈ *R*^*m*×*d*_*h*_^ and *L*_*att*_ ∈ *R*^*c*×*d*_*h*_^ denote the text representations of the label participation and text participation, respectively. The term *d*_*h*_ represents the hidden dimensions of the coordinate attention layer.

Furthermore, after IN, the residual connection and feedforward network (FN) obtain text and label fusion, encoding *X*_*fu*_ ∈ *R*^*m*×*d*^ and *L*_*fu*_ ∈ *R*^*c*×*d*^.

\begin{eqnarray*}\begin{array}{@{}l@{}} \displaystyle {X}_{fu}=I{N}_{X} \left( F{N}_{X} \left( {X}_{att} \right) +{X}_{enc} \right) ,\\ \displaystyle {L}_{fu}=I{N}_{L} \left( F{N}_{L} \left( {L}_{att} \right) +{L}_{enc} \right) . \end{array} \end{eqnarray*}

The FN maps the input to a higher dimension *d*, enabling mutual engagement between the text and label.

This study adopts a case normalization method that differs from that reported in a previous study (Liu et al., 2022) in the processing of text features. This method not only avoids dependence on a small range of neurons but also maintains the independence of text instances and accelerates the convergence speed of the model regardless of the channel or batch size. The normalization formula of IN is expressed in equations as follows:

\begin{eqnarray*}{y}_{tijk}= \frac{{x}_{tijk}-{\mu }_{ti}}{\sqrt{{\sigma }_{ti}^{2}+\in }} , \end{eqnarray*}

\begin{eqnarray*}{\mu }_{ti}= \frac{1}{HW} \sum _{l=1}^{W}\sum _{m=1}^{H}{x}_{tilm}, \end{eqnarray*}

\begin{eqnarray*}{\sigma }_{ti}^{2}= \frac{1}{HW} \sum _{l=1}^{W}\sum _{m=1}^{H}{ \left( {x}_{tilm}-m{u}_{ti} \right) }^{2}. \end{eqnarray*}

For the selection of the activation functions, GELU (Lee, 2023) were selected. GELU is a high-performance neural network activation function that has been successfully applied to the BERT model (Hendricks & Gimpel, 2016). GELU functions exhibit excellent generalization and stable optimization abilities, which can improve model performance and reduce the difficulty and time cost of model training. The mathematical expression of the GELU is reproduced as follows:

\begin{eqnarray*}GELUs(x)=xP(X\leq x)=x\Phi (x), \end{eqnarray*}

where Φ(*x*) denotes the probability function of a normal distribution.

To fully exploit the information encoded by the text engaged by the label and the relevance encoded by the label engaged by the text. In this study, a fusion encoder layer was introduced into the model and two mutually independent BiLSTM layers were constructed to propagate the fused text and label information. One BiLSTM layer was used to generate the ultimate text representation *X*_*fin*_ by combining the text encoding *X*_*fu*_ as follows:

\begin{eqnarray*}{X}_{fin}=BL{M}_{X} \left( {X}_{fu} \right) , \end{eqnarray*}

where *X*_*fin*_ ∈ *R*^*m*×*d*^. The subsequent decoding process is preserved in the hidden state *h* ∈ *R*^*d*×1^ and cell state *c* ∈ *R*^*d*×1^ of *BLM*_*X*_. These are utilized to initialize the hidden and cell states of the LSTM decoder, which assists in generating a logical output from the input sequence.

Another BiLSTM encoder fuses the label encoding to produce a sequence of the label *L*_*fin*_ ∈ *R*^*c*×*d*^ as follows:

\begin{eqnarray*}{L}_{fin}=BL{M}_{L} \left( {L}_{fu} \right) . \end{eqnarray*}

### ALD

For the final component of the model, we employed an ALD architecture. ALD consists of two steps for each time step. First, using the LSTM decoder, the hidden, cell, and loop context states were acquired in the first step of ALD decoding. Second, the probability of each class was determined using this component, which handles the classification problem simultaneously without modifying the model (Yang et al., 2018).

In the first step, we used LSTM cell attention as the benchmark technique for implementing the LSTM decoder. During training, we initially locate the label embeddings *e*_*t*−1_ of the $ \left( t-1 \right) $ decoding step in the true label and the predicted label embedding. We will use this alignment during the prediction process. Thereafter, the LSTM cell takes as input *e*_*t*−1_, the recurrent context state *r*_*t*−1_, the hidden layer state *h*_*t*−1_, and the previous time step of the cell state *c*_*t*−1_. It outputs the hidden state *h*_*t*_ and cell state *c*_*t*_ of the current time step *t*, as expressed in the following equation.

\begin{eqnarray*}{h}_{t},{c}_{t}=LSTMCell \left( \left[ {e}_{t-1};{r}_{t-1} \right] ,{h}_{t-1},{c}_{t-1} \right) , \end{eqnarray*}

where *h* and *c* denote the coding processes to initialize *h*_0_ and *c*_0_, respectively.

And then initialize *e*_0_ and *r*_0_. Once we obtained the hidden state *h*_*t*_, we calculated the result of *a*_*t*_ when the step size is *t*. The adaptive classifier receives the hidden state *h*_*t*_ for subsequent processing.

\begin{eqnarray*}{a}_{t}=Softmax \left( {X}_{fin}{W}_{1}{h}_{t} \right) , \end{eqnarray*}

where *W*_1_ denotes a trainable matrix, and *r*_*t*_ is the state of the context at time step *t*, as expressed in the following equation.

${r}_{t}=\mathrm{Tanh} \left( {W}_{2} \left[ {X}_{fin}^{T}{a}_{t};{h}_{t} \right] \right) $,

where *W*_2_ ∈ *R*^*d*×2*d*^ represents a trainable matrix.

We used an adaptive classifier in the final classification phase to utilize the label representation of information-text participation. In comparison with most existing methods, the adaptive classifier can directly output the probability of each class by focusing on the label representation of text participation.

### Label probability prediction

Given the hidden state *h*_*t*_ of the step size *t*, this classifier considers the final label of the code *L*_*fin*_ and the hidden state *h*_*t*_ as inputs to obtain the probability ${\hat {y}}_{t}$ of the time-step *t*.

\begin{eqnarray*}{\hat {y}}_{t}=Softmax \left( {L}_{fin}{W}_{3}{h}_{t} \right) , \end{eqnarray*}

where *W*_3_ represents a trainable matrix and ${\hat {y}}_{t}$ is the output probability in each class. Therefore, to simplify the classification process, we integrated label representation into the text-attention mechanism.

After obtaining the probabilities for all the categories, we optimized our model by computing the objective function.

## Results and Discussion

### Dataset

An experimental dataset called the COVID dataset was constructed by crawling COVID online reviews from December 2019 to March 2022 using Python Creeper technology. The constructed COVID dataset totaled 100,000 records. In the COVID dataset, Label 0 was designated as a negative sample, whereas Label 1 was considered a positive sample. The COVID dataset for the number of positive and negative sample distributions is shown in Table 1.

**Table 1:** COVID dataset distribution.

Labels	0	1
Number of instances	35,700	64,300

We also used Baidu’s AI open platform (https://ai.baidu.com) to conduct a series of early stages. First, data capture. Large amounts of data are collected from various online web pages. By adjusting parameters such as keywords, page numbers, results per page and domain Settings, Baidu ensures that comprehensive data is collected according to specific needs. Second, handle paging. To crawl multiple pages of search results, the crawl script systematically increases page parameters to cover the required number of pages. Finally, preliminary data cleaning. This includes deleting duplicate data and detecting errors.

To better analyze the data, further data cleansing was done, such as removing Spaces, URL address information, and hashtags. Doing so will prepare you for the subsequent preprocessing phase.

The purpose of the text preprocessing stage is to carry out a series of processing and conversion of the cleaned text in order to facilitate the subsequent development feature extraction and model training. Data preprocessing is divided into two steps. The first step is text segmentation. Using Jieba word segmentation method deals with the review data, which is of great help to some new words and words not included in the dictionary. Step two, build stop word list. This article refers to the common stop word list after adding a custom stop word, in order to better handle comments on the data. Each preprocessed COVID dataset included the comments and the corresponding sentiment label. The data is shown in part for example, see Table 2.

**Table 2:** Dataset partial sample table.

Label	Example
Positive	Now the sky, like a severe epidemic, envelops the city, and wishes the haze of the epidemic to withdraw as soon as possible.
Negative	What is wrong with people now, understanding that travel agencies want to make money, shouldn’t we advocate safe travel at this time.

### Evaluation indicators

We employed the micro-averaged F1 score (Mi-F1) to evaluate the performance of our model. The Mi-F1 is a common metric used in evaluating classification models, especially in scenarios where imbalanced class distributions are present. The micro-averaging method treats the contributions of all categories equally, making it suitable for datasets with imbalanced sample distributions, as it calculates overall performance by aggregating the results across all categories. In practical applications, the Mi-F1 pays more attention to categories with a large number of instances, which is particularly important in applications such as text classification. The calculation method of the Mi-F1 is straightforward, easy to understand, and implement, making it a widely adopted tool among researchers and practitioners.

For category *i*, true positives are denoted as *TP*_*i*_, false positives as *FP*_*i*_, and false negatives as *FN*_*i*_. The calculation formula is expressed as follows:

\begin{eqnarray*}Mi-F1= \frac{{\mathop{\sum \nolimits }\nolimits }_{i=1}^{c}2T{P}_{i}}{{\mathop{\sum \nolimits }\nolimits }_{i=1}^{c}2T{P}_{i}+F{P}_{i}+F{N}_{i}} . \end{eqnarray*}

### Model parameter setting

In this section, we concatenated all the labels in a randomly selected order. The resulting label sequence was identical for all samples in the dataset and included every label in the label set. Using the same label sequence for all the samples, the model learned to recognize and classify each label consistently across the entire dataset. This can enhance the performance and provide more reliable results. The experimental settings used in this study are listed in Table 3.

**Table 3:** Model parameter setting.

Experimental environment	Specific content
Word embedding	*d*_*emb*_ = 400
Internal model dimension	*d* = 256
BiLSTM output dimension	*d*/2
Hidden size of coordinate attention layer	*d*_*h*_ = 100
Attention headcount	3
Optimizer	Adam, the warmup schedule

### Experimental result

**Figure 2:**
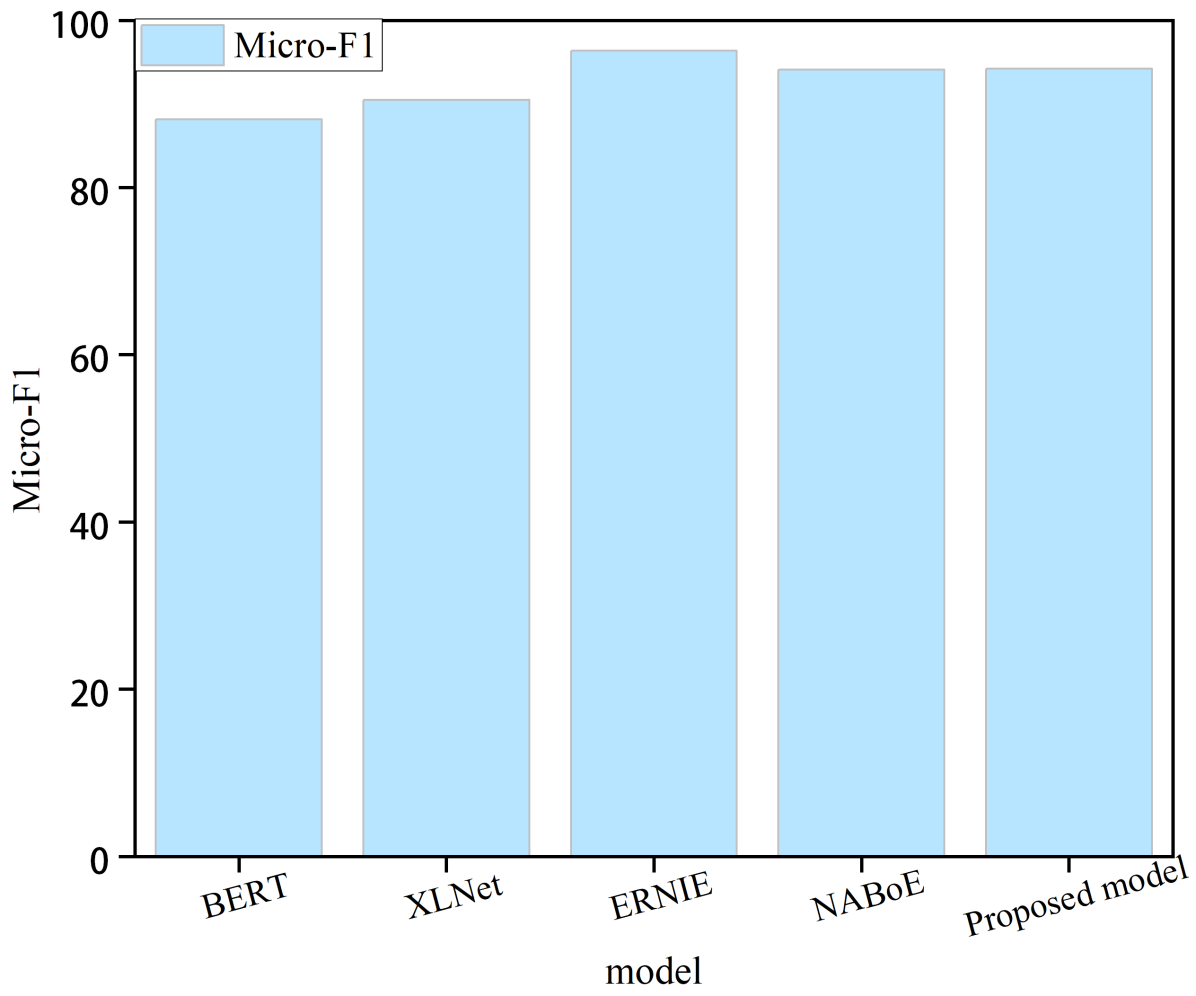
Comparison with the large pre-training model.

Large pre-training models, such as BERT (Devlin et al., 2018), XLNet (Yang et al., 2019), ERNIE 3.0 (Sun et al., 2021), and NABoE (Yamada & Shindo, 2019), have recently gained popularity and have also acquired powerful language representation capabilities using large-scale unsupervised corpora. Some of these models outperformed our model; however, the number of parameters in these pre-trained models was large, making them less computationally efficient. For instance, the ERNIE 3.0 pre-training model exhibited the best performance but with billions of parameters. The BERT pre-training model had relatively few parameters; however, the number of parameters reached 340 million. Figure 2 shows the performance of the model in this study compared with four large pre-training models. We can clearly observe that the proposed model exhibits certain shortcomings in terms of performance. The pre-training model with the lowest number of parameters had approximately 68 times the number of parameters of the proposed model. In real-world scenarios, where time and space constraints are critical, the sheer number of parameters required by pre-training models can pose a significant disadvantage. These models often require extensive computational resources and are difficult to deploy in practical applications with limited resources. Therefore, it is important to balance model performance with practical constraints when selecting a mode for a given task.

We conducted an experimental evaluation of the proposed model in comparison with several baseline models on the COVID dataset, and compares the time complexity of each model, the performance of the prediction algorithm in large-scale data and resource use efficiency has a larger advantage. Table 4 summarizes the results of this comparison.

**Table 4:** Comparison results.

Model	Mi-F1 score	Times
DocBERT	89.40	4 h
LSTM	86.06	2.5 h
VDCNN	90.21	3.8 h
HTTN	92.10	2.3 h
LEAM	91.75	2 h
LSAN	90.00	2 h
CNLE	93.47	1.8 h
Proposed model	94.18	1.7h

The DocBERT model (Adhikari et al., 2020) demonstrates superior performance in document classification by fine-tuning the BERT model.

The LSTM model (Chen, Tseng & Wang, 2021) possesses a sophisticated architecture that effectively manages long-term dependencies. Through the integrated function of its input, output, and forget gates, it dynamically controls the retention and omission of information within an LSTM unit at any given moment.

The Very Deep Convolutional Networks for Text Classification (VDCNN) (Conneau et al., 2016) employ minimal convolutional and pooling operations for character-based classification, enabling the model to incorporate 29 convolutional layers, thus facilitating deep-text classification.

The HTTN model (Xiao et al., 2021) introduces a novel head-to-tail network that capitalizes on the relationship between the head and tail labels to facilitate the transfer of meta-knowledge from data-rich tail labels to those with less data.

The LEAM model (Wang et al., 2018) innovatively embeds each label within the same vector space as the word vectors, utilizing an attention mechanism to gauge the compatibility between the text sequence and the label embeddings.

The LSAN model (Xiao et al., 2019) represents a label-specific attention network optimized for multi-label text classification tasks. It effectively exploits the semantic relationships between labels and words to enhance classification accuracy.

The CNLE model (Liu et al., 2022) deeply integrates sequence information from both labels and texts. This integration captures comprehensive representations by amalgamating input from both the text and labels, thereby enhancing the precision and comprehensiveness of the classification.

As shown in Table 4, label embedding-based methods (HTTN, LEAM, and LSAN) generally surpass text representation methods (DocBERT, LSTM, VDCNN), underscoring the effectiveness of label-oriented strategies in improving model performance.

The CNLE model utilizes labeled representations in conjunction with textual interactions, allowing for the labeling of text engagements and thereby enriching the data. This improvement is achieved through the IN method, which independently normalizes each sample, reducing variance across different input data distributions. This normalization significantly enhances the model’s ability to generalize across diverse datasets. Additionally, IN accelerates training convergence and improves stability by mitigating the Internal Covariate Shift (ICS)—the impact of input data distribution changes on model training. Therefore, the model described in this manuscript employs the IN method to enhance its generalization capabilities and stabilize the training process. Moreover, by integrating sequence information from both text and labels, IN helps the CNLE model process long textual data more effectively and capture contextual subtleties.

This study introduces the Gaussian Error Linear Unit (GELU) as the activation function in the proposed model to facilitate nonlinear improvements. The GELU function, by allowing the passage of small negative inputs, enables richer nonlinear transformations and the formation of more complex decision boundaries compared to the traditional ReLU. This is particularly beneficial in text categorization, where identifying subtle textual nuances is crucial. Within the CNLE framework, the GELU function enhances the interaction between text and label data, thus increasing the expressive power of the model’s features. This leads to more accurate text classification, especially in cases involving ambiguous or complex classification criteria.

In summary, the integration of the CNLE-based IN method and the GELU function significantly boosts the effectiveness of the text categorization model discussed in this paper. This approach is particularly effective in managing diverse and extensive textual data, markedly improving both the accuracy of classifications and the robustness of the model.

### Ablation studies

**Figure 3:**
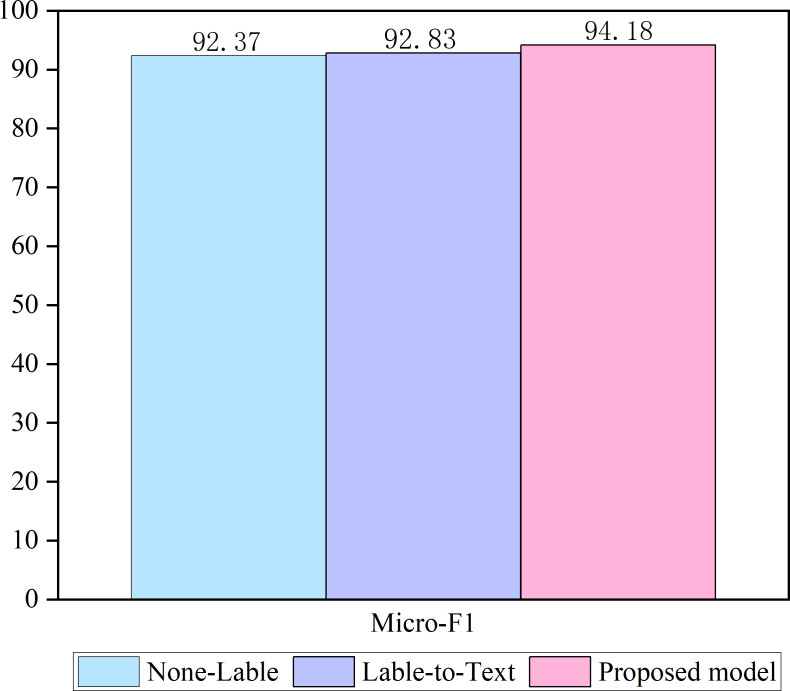
Ablation of the model.

Two ablation studies are conducted to test the validity of the proposed model. The results are shown in Fig. 3.

A none-labeled model was used in the first ablation study, where only a sequence of texts was used as the input to the model. Most of the existing models similarly approach this method. In the second ablation study, both label embedding and label text attention components were retained in our approach (referred to as label-to-text). However, text label attention has been eliminated in our approach, ensuring that no text is incorporated in the label embedding. Our approach incorporates a text-label coordinate attention architecture, and the ablation results demonstrate that using a label-attention text representation and text-attention label representation can effectively enhance classification accuracy. This finding highlights the importance of considering both text and label components and suggests that incorporating a coordinated approach between the two can enhance performance.

### Parameter analysis

In this section, we experimentally explored the effects of the hyperparameters on the overall performance of the proposed model. The hyperparameters involved in the experiment primarily included the number of attention heads in the multi-head attention layer and the size of the hidden layer in the coordinate attention mechanism.

The number of attention heads in the multi-head attention mechanism is an important hyperparameter, which determines the number of positions on which the model can focus. Increasing the number of attention heads can improve the expression and learning ability of the model such that the model can learn the relationship between different positions and features simultaneously to better capture the information of the input sequence. However, too many attention heads can lead to overfitting or performance degradation; therefore, an appropriate selection is required. The experimental results are listed in Table 5.

**Table 5:** Effect of the number of heads.

Attention headcount	Mi-F1 score
1	91.02
2	93.54
3	94.18
6	92.12
8	92.91

The data presented in Table 5 clearly illustrates that the performance of the proposed model is suboptimal when the attention head count is set to one. This diminished efficacy is attributable to the reduction of the multi-head attention mechanism to a single original attention model, which compromises the precise allocation of weight information across different positions. As the number of attention heads increases, there is a corresponding improvement in model performance, reaching an optimal state when the count is three. However, an escalation in the number of attention heads beyond this point results in a deterioration of performance, indicative of model overfitting. This phenomenon suggests that a higher number of attention heads may lead to excessive model complexity, which negatively impacts generalization.

Thereafter, we evaluated the effect of the hidden size of the coordinate attention mechanism, ranging from 100 to 300, on the performance of the model. The experimental results are listed in Table 6.

**Table 6:** Effect of the number of hidden sizes.

Hidden size	Mi-F1 score
100	94.18
200	93.68
300	93.05

As summarized in Table 6, the model performance gradually decreased as the size of the hidden layer in the coordinate attention mechanism increased. The decrease in performance is attributed to the increase in the dimension of word representation, which causes an increase in the training difficulty of the model, resulting in underfitting.

### Case study

In this section, experiments are conducted on two datasets, Yelp Polarity Reviews and Amazon Polarity Reviews, to further evaluate the model.

The Yelp Polarity Reviews dataset is extensively utilized in natural language processing and machine learning research, particularly for sentiment analysis. This dataset includes restaurant reviews from Yelp users, each annotated with either a positive or negative label, representing positive or negative sentiment respectively. It comprises over 500,000 reviews, evenly distributed between positive and negative sentiments.

Similarly, the Amazon Polarity Reviews dataset is frequently employed for sentiment analysis tasks. This dataset consists of product reviews from Amazon users, each labeled with positive or negative sentiment. It contains millions of reviews, providing a substantial resource for analyzing customer feedback and sentiment trends.

The experimental results of our proposed model for text classification on these two datasets are presented in Table 7.

**Table 7:** The performance of the model under different dataset.

Dataset	Mi-F1 score
Yelp polarity	75.50
Amazon polarity	81.70

Due to the lengthy training times required by deep learning models, the outcomes derived from various hyperparameters across different datasets will vary. Table 7 illustrates that the results achieved using the hyperparameters mentioned in the previous subsection are suboptimal for both datasets. To improve these results, extensive experimentation is necessary to further refine the model’s hyperparameters. However, this paper does not include extensive hyperparameter tuning experiments for these datasets.

## Conclusion

In this study, an optimized coordinate attention mechanism model was proposed to classify positive and negative samples to build a more efficient text classification model. First, we used label-attention text representation and text-attention label representation to obtain a shared representation of text and label sequences. By combining information from the text and labels, the model emphasized the relevant segments of both to a greater extent to perform text classification tasks better. Second, based on the self-attention model, IN and GELU functions were used, the convergence of the model was accelerated, and its performance was improved. Finally, using an adaptive decoder, we classified the comment text without modifying the model. Numerous experiments have indicated that the performance of the proposed method surpasses previous standard approaches.

This study was limited to the binary classification task of text and did not consider the needs of each scenario. Therefore, in the future, we will study the correspondence between labels in several task scenarios, apply them to multilabel classification tasks, and explore their advantages and disadvantages. It is also possible to extend the concept of coordinated attention mechanisms from text classification to other natural language processing tasks, such as natural language reasoning, dialogue systems, and language translation. By implementing such a mechanism, the performance of these NLP tasks can be improved by enabling the model to better capture the dependencies. We hope that the proposed method will promote research on text classification tasks in natural language processing and other fields, or consider adding discussion of potential limitations and future work to provide a balanced view of research contributions and areas of further investigation.

## Supplemental Information

10.7717/peerj-cs.2240/supp-1 Supplemental Information 1 Experimental data and code associated with the experiments performed Contents include:
1) data/: Directory likely containing datasets or scripts related to data handling.
2) networks/: Directory likely containing neural network architecture definitions and related files.
3) attn_network.py: Implements attention-based neural network architectures.
4) data_prepare.py: Prepares and processes data for training and evaluation.
5) evaluator.py: Evaluates the performance of trained neural network models.
6) metrics.py: Defines various metrics for model evaluation.
7) reader.py: Contains functions to read and load data.
8) score_ranges.py: Defines score ranges for evaluation metrics.
9) smart_open−6.3.0.tar: A tarball file likely containing the smart_open library for efficient streaming of large files.
10) utils.py: Utility functions to support various operations across the project.
11) utils.pyc: Compiled Python file for utils.py.

## Additional Information and Declarations

### Data Availability

The following information was supplied regarding data availability: Data and code are available in the Supplemental Files.
